# Inflammation as a key mediator: linking triglyceride-glucose index to prognosis in non-muscle-invasive bladder cancer

**DOI:** 10.3389/fonc.2025.1545985

**Published:** 2025-05-23

**Authors:** Yan Zhang, Zixiang Li, Xianfeng Shao, Shan Jiang, Jiahao Wang, He Zhang, Li Chen, Li Ding, Kun Wang, Wentao Xia, Junqi Wang

**Affiliations:** Department of Urology, The Affiliated Hospital of Xuzhou Medical University, Xuzhou, Jiangsu, China

**Keywords:** TyG index, bladder cancer, metabolic syndrome, inflammation, prognosis, tumour recurrence

## Abstract

**Background:**

Bladder carcinoma (BCa) is a prevalent urological malignancy characterised by high recurrence and progression rates, posing significant challenges in clinical management. The triglyceride-glucose (TyG) index has emerged as a promising prognostic marker for metabolic health in various cancers. This study explores the prognostic value of the TyG index in non-muscle-invasive bladder cancer (NMIBC), with a focus on its association with high-grade recurrence-free survival (RFS) and progression-free survival (PFS) and the mediating role of systemic inflammation.

**Methods:**

A total of 230 patients diagnosed with NMIBC between October 2017 and October 2022 were included in this retrospective study. Clinical and pathological data were collected alongside follow-up treatment outcomes. Mediation analysis was conducted to quantify the role of systemic inflammation, using markers such as C-reactive protein (CRP), interleukin-6 (IL-6), and interleukin-8 (IL-8), in the relationship between the TyG index and patient prognosis.

**Results:**

The TyG index was identified as a significant, non-linear prognostic factor for both RFS and PFS. An inverted U-shaped relationship was observed, with inflexion points at 9.186 and 9.168 for RFS and PFS, respectively. Below these thresholds, the TyG index was positively associated with worse outcomes (RFS: HR = 3.37, 95% CI = 1.77–6.41, *P* < 0.001; PFS: HR = 3.54, 95% CI = 1.65–7.58, *P* = 0.001). Mediation analysis revealed systemic inflammation as a critical intermediary, contributing significantly to the observed associations.

**Conclusion:**

These findings suggest that the TyG index could serve as a valuable tool for risk stratification and prognostic assessment in NMIBC. Its integration into clinical decision-making frameworks may improve personalised management strategies, particularly by targeting systemic inflammation as a modifiable factor.

## 1 Introduction

Bladder cancer (BCa) is among the malignant tumours with a poor prognosis worldwide, primarily owing to its high risks of recurrence and progression (1). Initially, approximately 70–75% of patients are diagnosed with non-muscle-invasive bladder cancer (NMIBC) (2). Despite the implementation of standard therapies such as transurethral resection of bladder tumours (TURBT) with intravesical instillation (IVI) therapy, tumour relapse or progression to the more aggressive muscle-invasive bladder cancer (MIBC) occurs in 30–80% of patients (3, 4). Despite the identification of numerous biomarkers for predicting NMIBC prognosis, these advancements have not yet resulted in marked improvements in overall survival rates for BCa patients (5, 6). This highlights the critical need for further investigation into the biological and clinical characteristics of NMIBC, as well as the discovery of novel biomarkers, which are essential for developing more effective therapeutic strategies.

Metabolic syndrome (MetS), characterised by dyslipidemia, hypertension, diabetes, and abdominal obesity (7), is increasingly considered a notable risk factor that adversely affects the outcomes of BCa patients (8, 9). Insulin resistance (IR) serves as a key pathogenic link between metabolic disturbances and oncogenesis, significantly contributing to tumour initiation and progression (7, 10). Extensive research has shown that IR elevates the risk of several carcinomas, like gastric, prostate, and non-small cell lung cancers, and is strongly associated with poorer prognoses (11, 12). A study by Montella M. et al. notably identified that IR is more pervasive among BCa patients than those without (13). Clinically, IR and lipid metabolism disorders are commonly assessed using the TyG index (14, 15), with the TyG index showing superior predictive value compared with Euglycaemic-Hyperinsulinaemic Clamp (clamp-IR) (16). Recent research has underscored the TyG index as a valuable marker for predicting gastric, renal, and prostate cancer outcomes (12, 17–19). However, the TyG index has not been explored in relation to its prognostic significance in NMIBC. It is hypothesised that an increased TyG index is believed to be strongly linked to a negative outcome in NMIBC patients.

Inflammation is a key factor in mediating chronic cellular damage and promoting tumour progression, and it is closely linked to the pathophysiology of IR (20, 21). Research has demonstrated that individuals with a higher TyG level tend to exhibit a heightened inflammatory state (20). Furthermore, the TyG index has been found to correlate with the systemic immune-inflammation index (SII), indicating a possible connection between metabolic dysfunction and systemic inflammation (22). The intricate and bidirectional relationship between inflammation and cancer has significant implications for NMIBC prognosis (23, 24). Together, these findings underscore the critical role of inflammation in cancer progression and suggest that the TyG index may further influence outcomes in BCa patients by exacerbating inflammatory pathways.

Building upon the background provided, this study hypothesizes that the TyG index significantly influences the prognosis of NMIBC patients by modulating systemic inflammation. Furthermore, inflammatory markers like interleukin-6 (IL-6) and interleukin-8 (IL-8) are proposed to mediate the association between the TyG index and patient outcomes. This study aims to thoroughly investigate the prognostic relevance of the TyG index among the NMIBC population, particularly emphasizing the mediating role of inflammation.

## 2 Materials and methods

### 2.1 Study design and population

**Figure 1:**
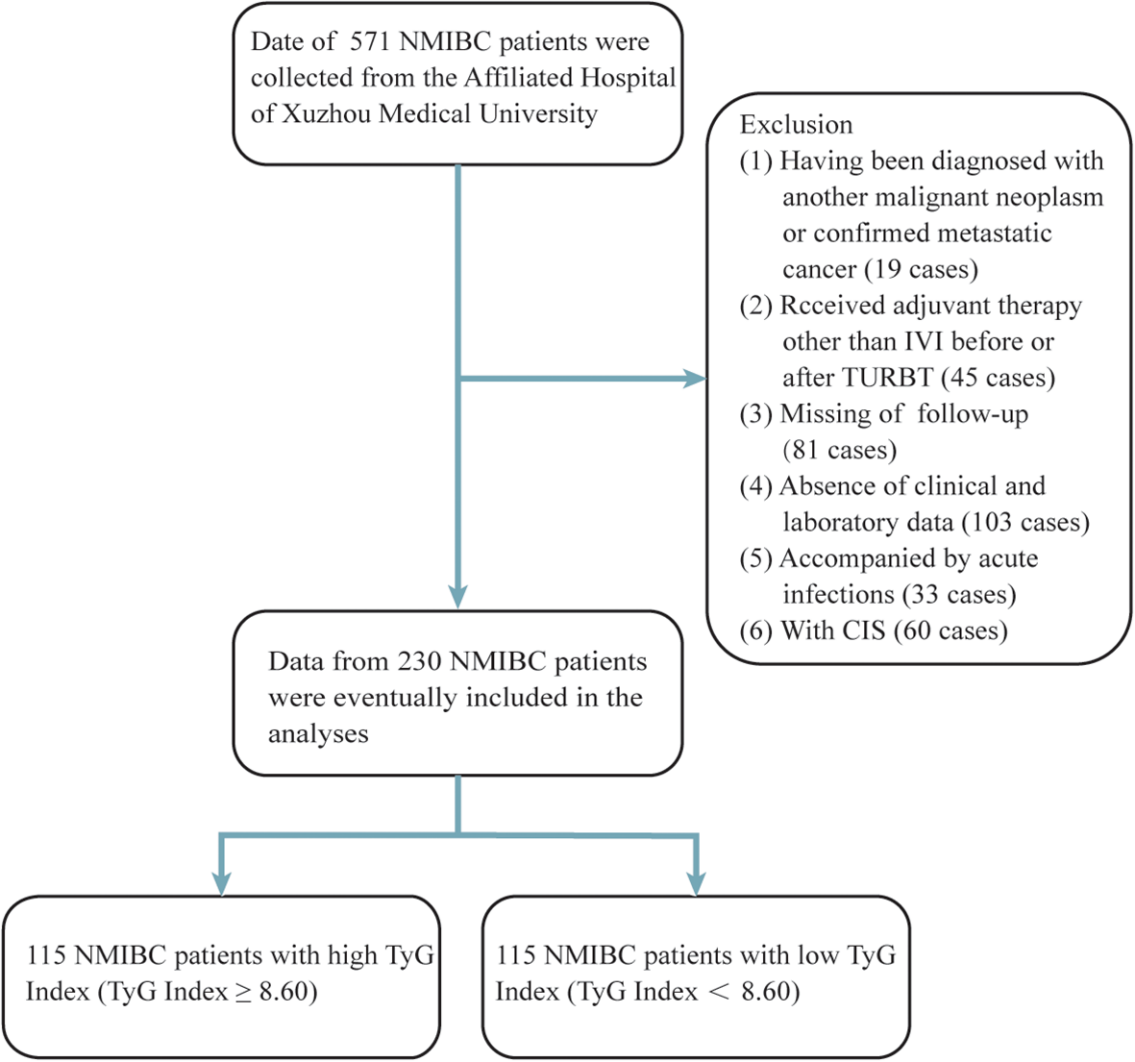
Process of clinical data collection and selection. NMIBC, non-muscle-invasive bladder cancer; IVI, intravesical instillation; TURBT, transurethral resection of the bladder tumour.

In accordance with ethical standards, written informed consent was waived due to the retrospective design of the study. Data from patients diagnosed with NMIBC between October 2017 and October 2022 were reviewed. The inclusion criteria were as follows: (1) TURBT as the surgical method; (2) pathological confirmation of urothelial carcinoma; and (3) clear postoperative pathological staging and grading with no evidence of muscle invasion or metastasis. Exclusion criteria included: (1) incomplete clinical data; (2) a diagnosis of other cancers before or after surgery; (3) treatments other than bladder instillations prior to or after TURBT; (4) preoperative acute infections; and (5) the presence of carcinoma *in situ* (CIS). Of the 571 NMIBC patients initially identified, 230 were ultimately included in the analysis (**Figure 1**).

### 2.2 Variables and definitions

This study collected a comprehensive range of clinical and pathological data, including details on postoperative treatment and follow-up outcomes. Demographic information, such as age and sex, was recorded, alongside key clinical parameters like hypertension, diabetes, and smoking status. Lipid metabolism markers, including fasting plasma glucose (FPG), high-density lipoprotein cholesterol (HDL-C), triglycerides (TG), and body mass index (BMI), were also documented. Inflammation-related markers, such as C-reactive protein (CRP), IL-6, IL-8, platelet count (Plt), and the C-reactive protein-albumin-lymphocyte (CALLY) index, were assessed. All haematological data were obtained from blood tests performed within one week prior to surgery to ensure accuracy and relevance to the patient’s health status. Tumour-specific characteristics, including tumour count, maximum tumour diameter, recurrence after TURBT, and postoperative pathological staging and grading, were also recorded. Pathological staging adhered to the 2017 TNM classification system for bladder tumours, lymph nodes, and metastasis, while histological grading followed the World Health Organization (WHO) criteria (2004/2016).

The TyG index was calculated using the formula: TyG = ln [TG (mg/dL) × FBG (mg/dL)/2], with a threshold value of 8.60, determined by the median (**Supplementary Figure 1**). Consistent with the principle of maximizing survival differences, the cut-off point for the TyG index is also set at 8.60, as indicated by the highest Youden index (**Supplementary Figure 2**). Based on this threshold, patients were divided into two groups: those with a TyG index ≥ 8.60, indicative of a higher risk of MetS and related complications, and those with a TyG index < 8.60.

Inflammatory markers, serving as mediating variables in this study, primarily included CRP, IL-6 and IL-8. Participants were classified based on their CRP levels, with values greater than 5 mg/L considered as high CRP and values of 5 mg/L or lower as low CRP. The X-tile tool was used to categorize IL-6 levels into two groups: high IL-6 (IL-6 > 13 pg/mL) and low IL-6 (IL-6 ≤ 13 pg/mL). Similarly, IL-8 concentrations were divided into high IL-8 (IL-8 > 4.6 pg/mL) and low IL-8 (IL-8 ≤ 4.6 pg/mL) groups. Additionally, the CALLY index was utilized as an indicator of systemic inflammation. CALLY index was calculated using the formula: CALLY index = Albumin(g/L) × Lymphocyte (×10^9/L)/[CRP (mg/L) ×10].

### 2.3 Patient follow-up

In accordance with follow-up guidelines for NMIBC, the clinical endpoints were recurrence-free survival (RFS) and progression-free survival (PFS). RFS was defined as the time from TURBT to the detection of high-grade recurrence, metastasis, or cancer-related death, while PFS referred to the time from surgery to clinical progression, metastasis, or cancer-related death. Patient monitoring was designed to track these endpoints. For low-risk patients, the first postoperative evaluation typically occurs three months after TURBT, followed by annual assessments. In contrast, intermediate- and high-risk patients, who require more intensive monitoring, are evaluated every 3 to 6 months during the first two years, transitioning to annual follow-ups thereafter. Diagnostic procedures, including blood and urine tests, cystoscopy, and urinary cytology, are regularly performed to detect potential recurrences. In patients at elevated risk for recurrence or disease progression, additional imaging via CT or MRI is employed to exclude the presence of invasive disease.

### 2.4 Adjuvant intravesical instillation therapy

With the exception of a few patients who declined bladder instillation, all others received their first instillation within 24 hours of TURBT. According to clinical guidelines, patients were advised to undergo weekly IVI for 6 to 8 weeks following TURBT, followed by monthly instillations for up to 12 months. Based on the patient’s postoperative bladder perfusion chart, the drugs of interest in this study included epirubicin, pirarubicin, gemcitabine, and Calmette-Guérin (BCG). In this study, the number of postoperative IVI was categorized into three groups: 0-1, 2-12, and more than 12, and this categorization was included in the prognostic analysis.

### 2.5 Statistical analysis

All statistical analyses were conducted using R software. Descriptive statistics, including means, medians, proportions, and interquartile ranges (IQRs), were used to summarize the data. Various statistical tests were employed to compare demographic, haematological, pathological, and imaging characteristics between the two TyG index groups. For categorical variables, differences between groups were assessed using the chi-square test or Fisher’s exact test, depending on the sample size and distribution. Kaplan-Meier survival curves were generated to examine survival differences between the two TyG index groups. A Cox proportional hazards model was applied for both univariate and multivariate analyses. The univariate analysis evaluated the effect of each variable independently, while the multivariate analysis adjusted for potential confounders. Three models were developed: an unadjusted model (without covariates), Model 1 (adjusted for gender, age, and BMI), and Model 2 (adjusted for additional factors, including repeat TURBT, hypertension, diabetes, smoking status, previous recurrence, tumour characteristics, and laboratory markers). To investigate the relationship between the TyG index and RFS and PFS, restricted cubic splines (RCS) with four knots (at 0.05, 0.35, 0.65, and 0.95) were used to model potential non-linear associations. Penalized splines were applied to smooth the curves. For non-linear relationships, a piecewise Cox regression model was employed to assess the association between the TyG index and RFS/PFS on either side of the inflexion points.

A bootstrap approach with 5,000 iterations was applied to evaluate the mediating effect of systemic inflammation. This analysis aimed to determine whether systemic inflammation modulates the TyG index with RFS and PFS. CRP, IL-6 and IL-8 were included as mediators. Additionally, in light of the increasing recognition of novel markers of systemic inflammation, the CALLY index was also incorporated into the analysis. The study further assessed the mediating roles of CRP, IL-6, IL-8 and CALLY index on outcomes in NMIBC patients.

## 3 Results

### 3.1 Characteristics of patients

In this study, 571 patient records were reviewed, with 230 cases involved. The median age of individuals diagnosed with NMIBC was 69 years. These patients ranged in age from 24 to 94 years, with 193 (83.91%) males. Among the participants, 76 (33.04%) were hypertensive, 29 (12.61%) were diabetic, and 71 (30.87%) had a smoking history. Most cases involved multiple tumours (56.09%), high-grade tumours (67.39%), T1-stage tumours (50.43%) and first-time occurrences (77.83%). In terms of follow-up treatment, 44 patients (19.13%) underwent a repeat TURBT within 6 weeks postoperatively. A total of 40 patients (17.39%) either declined postoperative bladder instillation or received only one instillation within 24 hours post-surgery, 40 patients (17.39%) received 2–12 instillations, and 150 (65.22%) completed standard adjuvant IVI therapy. Comparative analysis indicated that individuals who had elevated TyG index tended to have a higher BMI, elevated lipid levels, increased platelet and neutrophil counts, as well as higher CRP, IL-8 and CALLY index (*P* < 0.05) (**Table 1**).

**Table 1:** Clinical characteristics & baseline demographics of NMIBC patients.

Variables	Overall (n=230)	TyG index		*P*-Value
		Low (n=115)	High (n=115)	
Age (year)	69.00 (62.00, 78.00)	70.00 (63.00, 78.50)	67.00 (58.00, 77.00)	0.082
BMI	25.10 (22.66, 27.45)	24.22 (21.80, 27.02)	25.78 (23.39, 27.68)	0.001
TC (mmol/l)	4.50 (3.83, 5.13)	4.30 (3.54, 5.04)	4.72 (4.05, 5.41)	<0.001
TG (mg/dl)	1.25 (0.94, 1.70)	0.96 (0.76, 1.15)	1.70 (1.46, 2.09)	<0.001
FBG (mg/dl)	5.20 (4.70, 5.96)	4.93 (4.56, 5.37)	5.64 (4.96, 6.96)	<0.001
HDL-C (mg/dl)	1.11 (0.92, 1.32)	1.20 (1.01, 1.46)	1.04 (0.83, 1.19)	<0.001
Hb (g/l)	139.00 (127.00, 148.00)	137.00 (124.00, 145.50)	142.00 (131.00, 150.00)	0.023
Alb (g/l)	42.95 (40.12, 45.18)	42.10 (39.40, 44.30)	43.40 (40.60, 46.55)	0.010
CRP (mg/dl)	2.10 (0.70, 6.90)	1.40 (0.50, 4.20)	3.20 (1.10, 8.00)	0.002
IL-6 (pg/ml)	3.72 (2.04, 10.30)	3.32 (2.05, 6.67)	4.89 (2.02, 15.30)	0.120
IL-8 (pg/ml)	2.44 (1.46, 7.67)	2.36 (1.20, 4.38)	3.18 (1.71, 8.71)	0.004
Platelet count (×10^9/l)	202.00 (167.00, 234.75)	196.00 (160.50, 231.50)	208.00 (171.00, 240.00)	0.046
Neutrophil count (×10^9/l)	3.38 (2.77, 4.38)	3.28 (2.69, 3.80)	3.69 (2.91, 4.77)	0.001
Lymphocyte count (×10^9/l)	1.80 (1.40, 2.30)	1.70 (1.30, 2.20)	1.80 (1.50, 2.30)	0.140
CALLY index	3.27 (1.02, 8.96)	4.29 (1.34, 15.25)	2.44 (0.91, 7.39)	0.037
Maximum tumor diameter (mm)	20.00 (15.00, 30.00)	22.00 (15.00, 30.00)	20.00 (15.00, 30.00)	0.333
Gender, n (%)				0.858
Female	37 (16.09)	19 (16.52)	18 (15.65)	
Male	193 (83.91)	96 (83.48)	97 (84.35)	
Tumor number, n (%)				0.894
Multiple	129 (56.09)	65 (56.52)	64 (55.65)	
Single	101 (43.91)	50 (43.48)	51 (44.35)	
Pathology grade, n (%)				0.888
High-grade	155 (67.39)	78 (67.83)	77 (66.96)	
Low-grade	75 (32.61)	37 (32.17)	38 (33.04)	
T category, n (%)				0.598
T1	116 (50.43)	56 (48.70)	60 (52.17)	
Ta	114 (49.57)	59 (51.30)	55 (47.83)	
Hypertension, n (%)				0.050
No	154 (66.96)	84 (73.04)	70 (60.87)	
Yes	76 (33.04)	31 (26.96)	45 (39.13)	
Diabetes, n (%)				0.010
No	201 (87.39)	107 (93.04)	94 (81.74)	
Yes	29 (12.61)	8 (6.96)	21 (18.26)	
Smoking status, n (%)				0.886
No	159 (69.13)	80 (69.57)	79 (68.70)	
Yes	71 (30.87)	35 (30.43)	36 (31.30)	
Prior recurrence status, n (%)				0.427
Primary	179 (77.83)	92 (80.00)	87 (75.65)	
Recurrent	51 (22.17)	23 (20.00)	28 (24.35)	
Repeat TURBT, n (%)				0.737
No	186 (80.87)	94 (81.74)	92 (80.00)	
Yes	44 (19.13)	21 (18.26)	23 (20.00)	
IVI times, n (%)				0.670
0-1	40 (17.39)	22 (19.13)	18 (15.65)	
2-12	40 (17.39)	18 (15.65)	22 (19.13)	
>12	150 (65.22)	75 (65.22)	75 (65.22)	

Median (IQR) is used to present continuous variables, while percentages are used to present categorical variables.

BMI, Body mass index; TC, Serum total cholesterol; TG, Triglyceride; FBG, Fasting blood glucose; HDL-C, High-density lipoprotein cholesterol; Hb, Hemoglobin; Alb, Albumin; CRP, C-reactive protein; IL-6, Interleukin-6; IL-8, Interleukin-8; SII, Systemic immune-inflammation index; IVI: intravesical instillation; TURBT, transurethral resection of bladder tumours.

### 3.2 Patient prognosis

**Figure 2:**
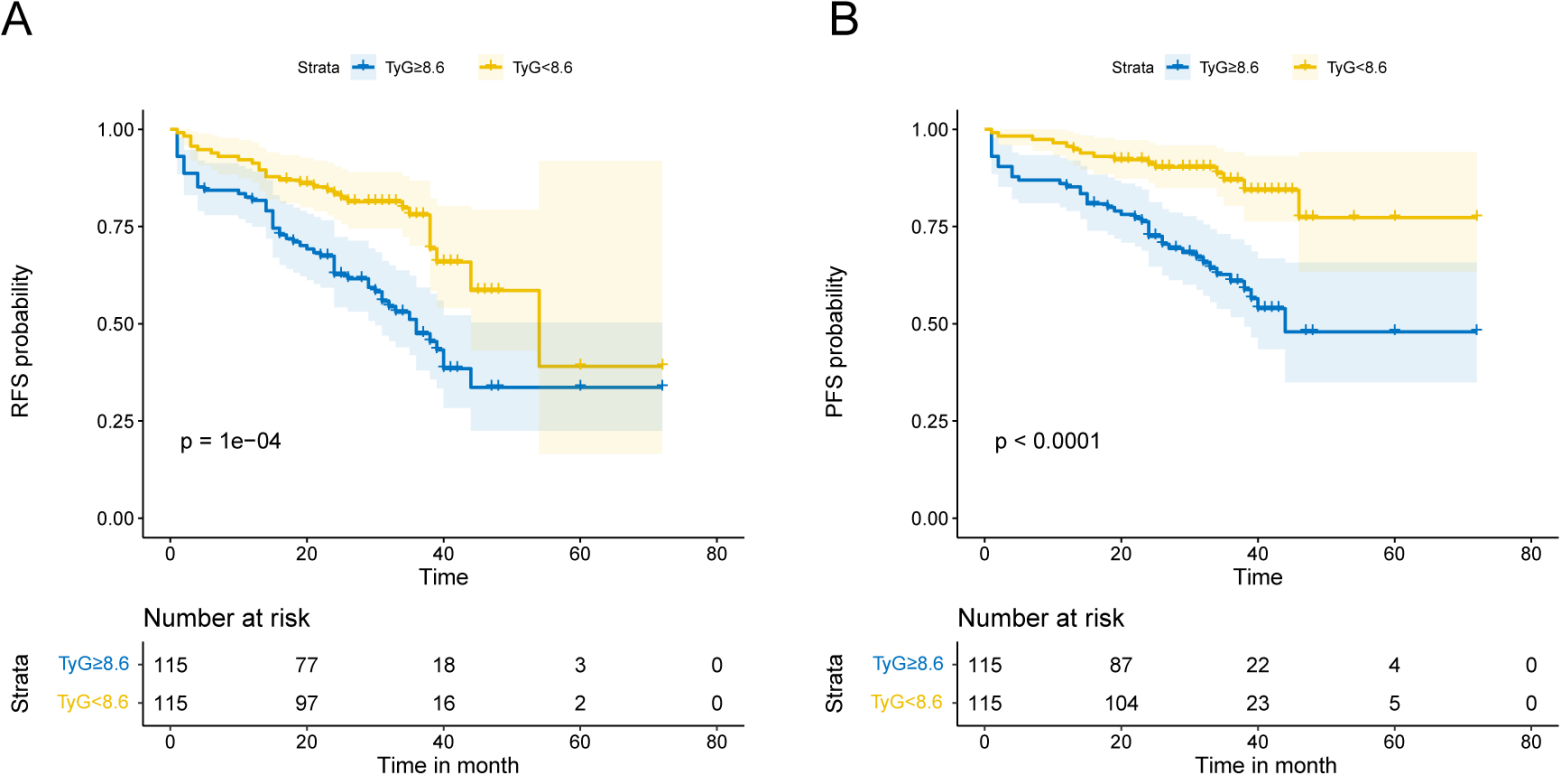
Kaplan-Meier analysis depends on the TyG index. Kaplan-Meier analysis with median TyG index as a cutoff point in terms of RFS **(A)** and PFS **(B)**.

Patients had varying lengths of follow-up periods, which spanned from 1 to 72 months. Patients were monitored for a median of 30.5 months, allowing for a comprehensive assessment of their outcomes over a significant period. During this period, 87 patients experienced high-grade recurrence, resulting in a recurrence rate of 37.82%, while 59 cases of progression were recorded, yielding a progression rate of 25.65%. RFS and PFS were evaluated through Kaplan–Meier survival curves (**Figure 2**), which revealed that the TyG index is negatively correlated with patient prognosis.

**Tables 2** and **3** present the association between the TyG index and RFS as well as PFS in patients with NMIBC. Overall, after adjusting for multiple variables, a statistically significant positive correlation was observed between the TyG index and both RFS (HR = 1.652, 95% CI = 1.015–2.689, *P* = 0.043) and PFS (HR = 1.905, 95% CI = 1.017–3.570, *P* = 0.044), indicating that higher TyG levels are associated with worse survival outcomes. Furthermore, when patients were stratified by the median TyG index, those in the high TyG group demonstrated notably worse survival outcomes. Specifically, the high TyG group had significantly lower RFS (HR = 2.280, 95% CI = 1.272–4.087, *P* = 0.006) and PFS (HR = 5.406, 95% CI = 2.383–12.261, *P* < 0.001) compared to the low TyG group. In conclusion, after adjusting for confounding variables, the TyG index, whether considered as a continuous or categorical variable, exerts a significant influence on the prognosis of NMIBC patients. Higher TyG index values are strongly associated with worse outcomes in both RFS and PFS, suggesting that a higher TyG index serves as a predictor of poorer prognosis in these patients.

**Table 2:** Relationship between TyG index and recurrence-free survival.

TyG index	Non-adjusted	*P*-value	Adjust I	*P*-value	Adjust II	*P*-value
Continuous	1.822 (1.296 - 2.560)	<0.001	2.015 (1.366 - 2.973)	<0.001	1.652 (1.015 - 2.689)	0.043
Group						
Low	1.000 (Reference)		1.000 (Reference)		1.000 (Reference)	
High	2.341 (1.498 - 3.658)	<0.001	2.638 (1.644 - 4.234)	<0.001	2.280 (1.272 - 4.087)	0.006

Non-adjusted model adjusted for: None. Adjust I model adjusted for: Gender, Age and BMI. Adjust II model adjusted for: Repeat TURBT, Gender, Hypertension, Diabetes, Smoking, Prior recurrence status, Tumor number, Pathology grade, T category, IVI times, Maximum tumor diameter, TC, HDL-C, Hb, Alb, Platelet count, Neutrophil count, Lymphocyte count, Age and BMI.

**Table 3:** Relationship between TyG index and progression-free survival.

TyG index	Non-adjusted	*P*-value	Adjust I	*P*-value	Adjust II	*P*-value
Continuous	1.873 (1.235 - 2.840)	0.003	2.090 (1.286 - 3.396)	0.003	1.905 (1.017 - 3.570)	0.044
Group						
Low	1.000 (Reference)		1.000 (Reference)		1.000 (Reference)	
High	3.430 (1.908 - 6.167)	<0.001	4.085 (2.195 - 7.601)	<0.001	5.405 (2.383 - 12.261)	<0.001

Non-adjusted model adjusted for: None. Adjust I model adjusted for: Gender, Age and BMI. Adjust II model adjusted for: Repeat TURBT, Gender, Hypertension, Diabetes, Smoking, Prior recurrence status, Tumor number, Pathology grade, T category, IVI times, Maximum tumor diameter, TC, HDL-C, Hb, Alb, Platelet count, Neutrophil count, Lymphocyte count, Age and BMI.

### 3.3 The non-linear relationship between the TyG index and prognosis in patients NMIBC

**Figure 3:**
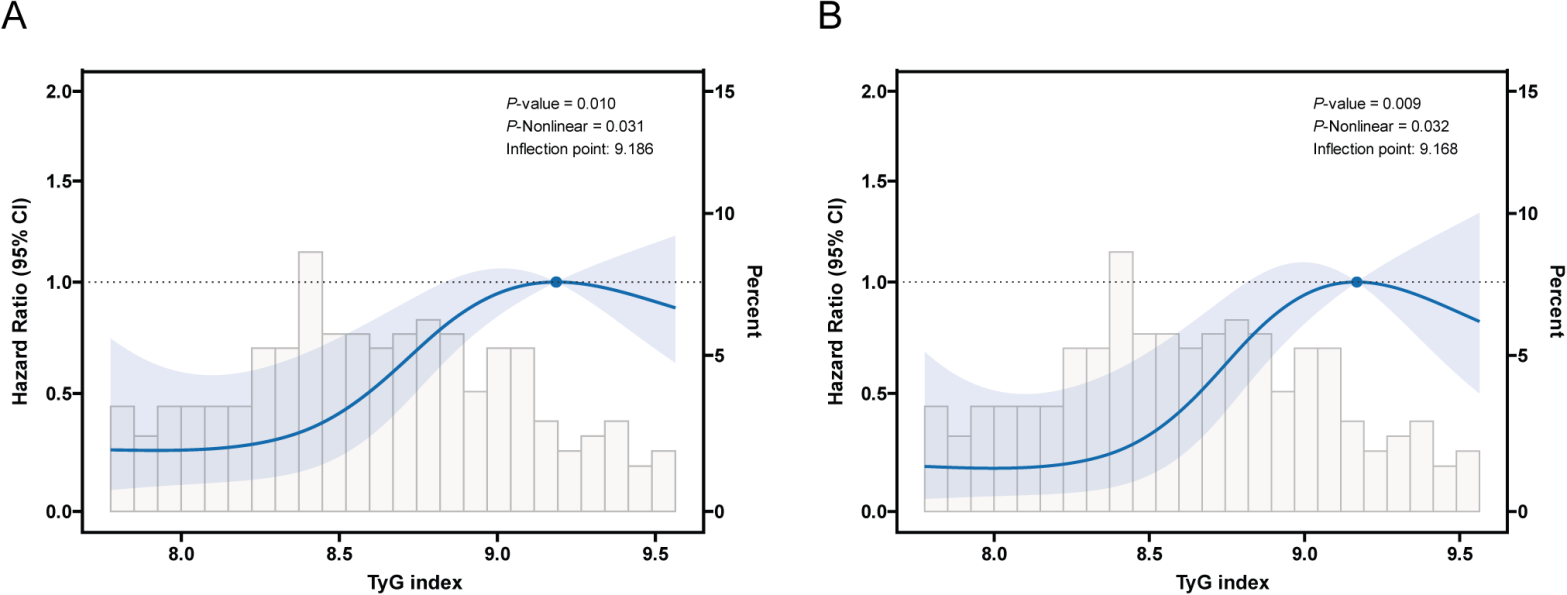
Restricted cubic spline (RCS) curve for the association of TyG index with RFS and PFS after adjusting for all variables. **(A)** Association between TyG index and RFS in NMIBC patients. **(B)** Association between TyG index and PFS in NMIBC patients.

As shown in **Figure 3**, after adjusting for multiple potential confounding factors, a non-linear relationship was observed between the TyG index and both RFS and PFS in patients with NMIBC. To further explore this relationship, RCS and segmented Cox regression were utilized. **Figures 3A, B** illustrate an inverted U-shaped association between the TyG index and both RFS and PFS, with optimal inflexion points at 9.186 and 9.168, respectively (**Table 4**). Below these inflexion points, an increase in the TyG index was associated with progressively higher risks for both RFS (HR = 3.37, 95% CI = 1.77 - 6.41, *P* < 0.001) and PFS (HR = 3.54, 95% CI = 1.65 - 7.58, *P* = 0.001). Beyond these thresholds, the association plateaued, with risk levels stabilizing.

**Table 4:** Threshold effect analysis of TyG index on recurrence-free survival and progression-free survival.

	Adjusted HR (95% CI)	*P*-value
RFS		
Inflection point	9.186	
TyG index < 9.186	3.37 (1.77 - 6.41)	<0.001
TyG index ≥ 9.186	1.71 (0.50 - 5.87)	0.39
*P* for Loglikelihood ratio	0.006	
PFS		
Inflection point	9.168	
TyG index < 9.168	3.54 (1.65 - 7.58)	0.001
TyG index ≥ 9.168	1.79 (0.38 - 8.52)	0.46
*P* for Loglikelihood ratio	0.005	

Cox proportional hazard models were used to estimate HR and 95% CI. Adjusted for: Repeat TURBT, Gender, Hypertension, Diabetes, Smoking, Prior recurrence status, Tumor number, Pathology grade, T category, IVI times, Maximum tumour diameter, TC, HDL-C, Hb, Alb, Platelet count, Neutrophil count, Lymphocyte count, Age and BMI.

### 3.4 Mediation effect of inflammation

**Figure 4:**
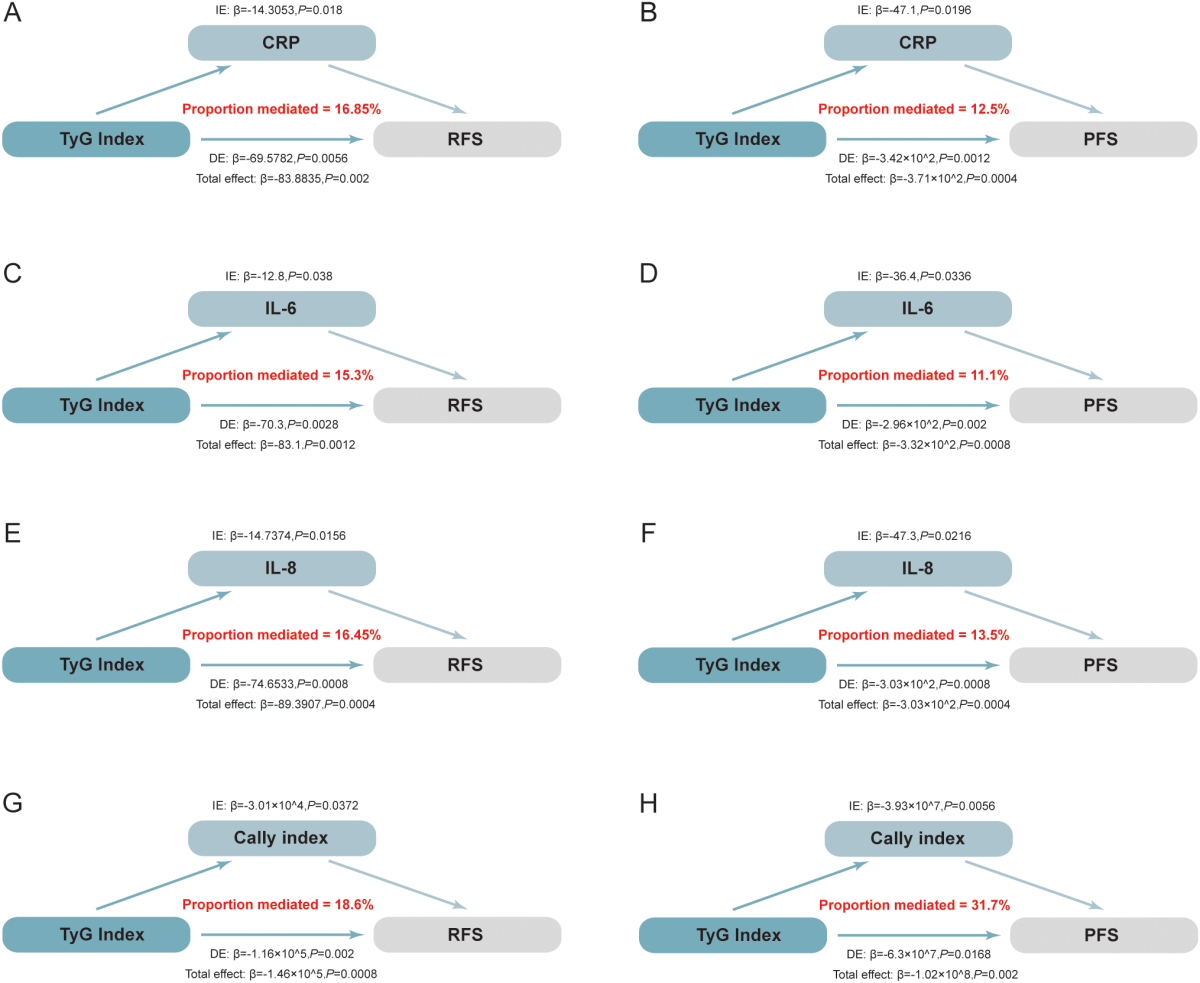
Mediation analysis illustrating the estimated proportion of the association between the TyG index and the prognosis of NMIBC mediated by inflammatory factors. The total effect is decomposed into direct and indirect effects, where the indirect effect quantifies the mediated proportion through inflammatory markers. Statistical significance and 95% CIs were estimated using bootstrap resampling (5,000 iterations). **(A)** The mediating effect of CRP on RFS: CRP mediated 16.85% of the total effect (*P*=0.018). **(B)** The mediating effect of CRP on PFS: CRP mediated 12.50% of the total effect (*P*=0.020). **(C)** The mediating effect of IL-6 on RFS: IL-6 mediated 15.30% of the total effect (*P*=0.038). **(D)** The mediating effect of IL-6 on PFS: IL-6 mediated 11.10% of the total effect (*P*=0.034). **(E)** The mediating effect of IL-8 on RFS: IL-8 mediated 16.45% of the total effect (*P*=0.016). **(F)** The mediating effect of IL-8 on PFS: IL-8 mediated 13.50% of the total effect (*P*=0.022). **(G)** The mediating effect of the CALLY index on RFS: The CALLY index mediated 18.60% of the total effect (*P*=0.037). **(H)** The mediating effect of the CALLY index on PFS: The CALLY index mediated 31.70% of the total effect (*P*=0.005). IL-6, Interleukin-6; IL-8, Interleukin-8; CALLY index, C-reactive protein-albumin-lymphocyte index; RFS, recurrence-free survival; PFS, progression-free survival; CRP, C-reactive protein;.

Mediation analysis revealed significant mediating effects of CRP, IL-6, IL-8 and CALLY index between the TyG index and RFS/PFS (**Figure 4**). Specifically, CRP, IL-6, IL-8 and CALLY index mediated 16.85%, 15.3%, 16.45% and 18.6% effect between the TyG index and RFS, respectively (*P* < 0.05) (**Figures 4A, C, E, G**). Similarly, these markers mediated a 12.5%, 11.1%, 13.5% and 31.7% effect between the TyG index and PFS (*P* < 0.05) (**Figures 4B, D, F, H**). A bootstrap test with 5,000 iterations confirmed the significance of these mediation effects, as the 95% CIs did not include zero. These results highlight the role of systemic inflammation as a key mediator between the TyG index and NMIBC individuals’ prognosis.

### 3.5 Subgroup analysis and interactions

**Figure 5:**
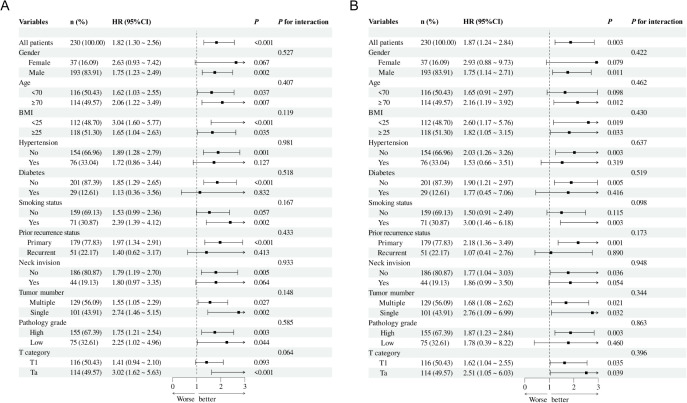
Subgroup analysis and interaction analysis based on RFS **(A)** and PFS **(B)**. BMI, body mass index.

An additional investigation was conducted into the correlation between NMIBC prognosis and the TyG index through subgroup analysis (**Figure 5**). This analysis revealed that RFS reached statistical significance among the following subgroups: males, no history of diabetes and hypertension, smoking experience, and Ta pathology (**Figure 5A**). Similarly, PFS reached statistical significance in the subgroups with no history of diabetes and hypertension, males, age more than 70 years, no bladder neck involvement, and high-grade pathology (**Figure 5B**). No notable interaction emerged between the two subgroups (total *P* values > 0.05).

## 4 Discussion

This single-center, real-world retrospective study is the pioneer in exploring the role of systemic inflammation as a mediator between the preoperative TyG index and NMIBC prognosis. Systemic inflammatory markers, including CRP, IL-6, IL-8 and CALLY index, were identified as key mediators between the TyG index and NMIBC patients’ prognosis. Notably, IL-8 mediated 16.45% of the effect on RFS and 13.5% on PFS, highlighting the significant impact of inflammation on patient outcomes. This study demonstrates that a higher preoperative TyG index is associated with worse outcomes, and underscores the critical role of systemic inflammation as a mediator in NMIBC prognosis.

IR, a key component of MetS, is a key player in the progression of malignant tumours (11, 12). The TyG index, a newly introduced and easily measurable index, has shown superior predictive value. Owing to its simplicity and effectiveness, the TyG index has replaced traditional indices of IR and is regarded as an important risk factor for several tumours (14, 15, 25). Several studies have established this connection across different cancer types (12, 18). For instance, as recognized by Tong Liu et al., there is a crucial relationship between colorectal cancer and high TyG index levels (26). Similarly, the TyG index has been found that it can independently predict prostate cancer in patients after prostate biopsy (25). Elevated TyG levels have been linked to a higher likelihood of developing breast cancer (27). Further extending these findings, recent research has underscored the TyG index as a promising marker for assessing clinical progression in gastric, renal, and prostate cancers (17, 19). The TyG index has been linked to greater tumour aggressiveness and progression in renal cancer. In prostate cancer, hyperinsulinemia, a condition strongly associated with the TyG index, has been identified as a contributor to the increased incidence of the disease (18). The observed relationship between elevated TyG index levels and poorer prognosis in NMIBC reflects similar findings in other cancers, highlighting the potential of the TyG index as a universal marker in tumour biology across multiple malignancies. Additionally, some studies have revealed that IR is more pervasive among BCa patients than among those without (13). Giovanni Tarantino et al. found individuals with BCa displayed significantly elevated serum TyG levels relative to individuals without the condition (28). However, there is still a research gap in the TyG index along with the endpoints of BCa individuals. This presence of this gap underscores the need for a comprehensive study aimed at elucidating the prognostic significance of the TyG index among NMIBC populations.

Survival analysis revealed significantly higher recurrence and disease progression rates in the high TyG group, with the cutoff defined at the median value of 8.6. The TyG index, as a representative biomarker closely linked to MetS (14–16), suggests that NMIBC patients with MetS may have a worse prognosis. This finding aligns with previous studies connecting MetS to poorer cancer outcomes (9, 29). Furthermore, after adjusting for confounding variables, this study observed a distinct inverted U-shaped relationship between the TyG index and both RFS and PFS in NMIBC patients. This suggests a non-linear effect of the TyG index on patient prognosis. Previous studies have similarly demonstrated that the TyG index exhibits a non-linear relationship with tumour development and prognosis in various populations. For instance, Xueyan Wu et al. found a J-shaped association between the TyG index and breast cancer incidence and prognosis, providing further evidence of the non-linear role of the TyG index in cancer prognosis (30).

The mechanisms linking the TyG index to BCa prognosis remain unclear but are likely mediated by multiple interconnected pathways, including inflammation, oxidative stress, and hyperinsulinemia (20, 31, 32). Among these, inflammation is considered a key driver (31). MetS, often resulting from excess fat accumulation, induces a pro-inflammatory state (33). Fat tissue operates as an endocrine organ, releasing adipokines. In MetS, this secretion is dysregulated, leading to an overproduction of pro-inflammatory cytokines, like IL-18 and TNF-α (34). This creates a cycle of chronic inflammation, which may promote tumour progression. Christina M. Vidal et al. confirmed that MetS is closely associated with the senescence-associated secretory phenotype (SASP), a process that generates inflammatory factors, creates an inflammatory microenvironment, and accelerates tumour angiogenesis, invasion, and metastasis (31). This suggests that the chronic inflammation linked to MetS could be a critical factor connecting the TyG index to BCa outcomes. Furthermore, free fatty acids (FFAs) can intensify inflammation by engaging Toll-like receptor 4 (TLR-4), initiating downstream inflammatory signaling pathways (35, 36). This process is essential, as research has shown that higher TyG levels correlate with enhanced TLR-4 expression. TLR pathway activation may thus represent a key mechanism through which the TyG index promotes inflammation and poor BCa prognosis. Furthermore, Koenen et al. demonstrated that elevated triglyceride and FFA levels in obese individuals induce pyroptosis, a type of programmed cell death marked by the enlargement and demise of adipocytes, which subsequently produces reactive oxygen species (ROS) and activates the NLRP3 inflammasome (37). This process perpetuates IR and creates a cycle of inflammation, thereby worsening metabolic dysregulation and potentially contributing to poor BCa outcomes. Therefore, this study conducted a mediation analysis to elucidate the key role of systemic inflammation as a mediator between the TyG index and RFS/PFS in NMIBC patients. By analyzing the mediatory effects of CRP, IL-6, IL-8, and the CALLY index, it was shown that these inflammatory mediators may be significant contributors to the poor prognosis observed in NMIBC patients.

CRP, a well-established acute-phase reactant, increases during inflammatory responses and plays a key role in modulating immune functions (38, 39). In NMIBC, elevated CRP levels are thought to worsen systemic inflammation, impair immune responses, and contribute to tumour progression (39). IL-6, a cytokine that regulates immune functions and promotes tumorigenesis, further mediates this relationship by fostering a pro-inflammatory environment that supports cancer cell growth and survival (39, 40). Similarly, IL-8, another pro-inflammatory cytokine, is implicated in tumour angiogenesis, metastasis, and immune evasion, all of which are associated with poorer clinical outcomes in NMIBC (34, 41).Consequently, these inflammatory markers highlight the critical role of systemic inflammation in associating the TyG index with poor prognosis in NMIBC. This underscores the substantial impact of inflammation on tumor progression and patient survival.

MetS is a key driver of tumour initiation and progression, mediated not only by inflammation but also by oxidative stress and hormonal dysregulation. Oxidative stress, predominantly caused by lipid peroxidation, elevates ROS levels, resulting in cellular and DNA damage, promoting mutations, and accelerating cellular senescence (37). These processes collectively contribute to tumour growth. Additionally, IR, a hallmark of metabolic syndrome, leads to hyperinsulinemia, which activates insulin-like growth factor-1 (IGF-1) receptors and initiates downstream signalling cascades that promote cell proliferation (42). Further compounding these effects, hyperglycemia aggravates oxidative stress by impairing mitochondrial function, reducing DNA repair efficiency, and amplifying ROS production (43). Collectively, these interconnected mechanisms, including oxidative stress, hormonal dysregulation, and inflammation, synergistically create a tumour-promoting microenvironment in NMIBC patients with metabolic syndrome, accelerating disease progression.

Recent studies have also indicated that interventions targeting inflammation and lipid metabolism could improve outcomes. For example, statin therapy has been associated with significantly enhanced survival outcomes in NMIBC patients receiving BCG bladder instillation (44). An animal study by Belmiro Parada et al. revealed that atorvastatin suppresses BCa progression in mice via its anti-inflammatory and antioxidant effects (45). Although further clinical evidence is needed, these findings suggest that targeting abnormal lipid metabolism and inflammation could offer therapeutic benefits for BCa patients.

The TyG index has demonstrated significant potential as a biomarker for systemic MetS and inflammation in NMIBC. Unlike other emerging biomarkers, such as the urobiome and circulating tumour markers, the TyG index is distinctive in its ability to concurrently reflect both metabolic disturbances and chronic inflammation. This unique feature makes it a valuable tool in clinical practice, particularly for large-scale screening. Studies, such as those by Carmela Nardelli and colleagues, have identified Porphyromonas somerae as a species associated with tumour progression (46). Additionally, microbial dysbiosis has been linked to a pro-inflammatory tumour microenvironment, underscoring the potential role of urobiome analysis in early cancer detection and prognosis. In contrast, circulating tumour biomarkers offer dynamic monitoring of tumour load but are limited by high costs and complex procedures, restricting their widespread application (47). While the TyG index is easily accessible and practical, it has limitations in providing localized or real-time insights into tumour dynamics. Therefore, future research should investigate integrating the TyG index with other biomarkers, such as urobiome analysis, to develop more robust predictive models that could improve the clinical management of NMIBC and enhance early detection strategies.

This study has significant clinical implications for managing NMIBC patients. Effective strategies such as low inflammation, low-fat diets, weight management, and increased physical activity can significantly reduce chronic inflammation. These interventions assist in managing the TyG index and addressing the metabolic imbalances linked to MetS. High-risk patients can then implement lifestyle modifications and adopt anti-inflammatory diets prior to TURBT, followed by rigorous postoperative monitoring for recurrence. For those unable to manage their metabolism through diet alone, statin therapy may be considered to correct metabolic abnormalities and enhance long-term outcomes.

This real-world study strengthens the reliability of its findings through rigorous preoperative evaluations, treatment protocols, and comprehensive prognostic assessments. The inclusion of extensive clinical data and subgroup analyses provides valuable insights into the prognosis of diverse patient populations, further supporting the study’s conclusions. However, several limitations should be acknowledged. First, the retrospective, single-center design introduces potential selection bias. The limited geographic and demographic diversity of the patient population reduces the generalizability of the results. To enhance external validity, future studies should incorporate diverse ethnic groups and adopt multi-centre designs to broaden population representation. Second, while the study identifies associations between the TyG index and RFS and PFS, it does not address the impact of temporal changes in the TyG index. Future research should explore these dynamic variations and evaluate the TyG index as a dynamic predictor of long-term outcomes. Additionally, patients who have addressed metabolic abnormalities through medication may experience changes in their TyG index, which could affect its predictive accuracy. Future studies should consider these factors to ensure the TyG index remains a reliable prognostic marker across various treatment contexts. Third, although this study proposes the TyG index as an independent prognostic factor, it was not directly compared with established NMIBC risk models such as the EORTC risk tables due to the absence of certain key clinical variables in our dataset. This limits the evaluation of its additive or comparative predictive performance. Future research should aim to incorporate these standard prognostic models to assess the complementary value of the TyG index. Fourth, the prognostic findings have not yet been validated in an external cohort. While the current study provides important preliminary evidence, external validation using multicenter and demographically heterogeneous datasets will be essential to confirm the robustness and clinical applicability of the results. Fifth, although we excluded patients with overt acute infections or known inflammatory disorders, systemic inflammatory markers such as CRP, IL-6, and IL-8 remain nonspecific and may still be influenced by unmeasured confounders. Future studies should include more comprehensive clinical profiling or additional inflammatory biomarkers to reduce residual confounding in mediation analysis. Sixth, while we discussed the potential clinical implications of targeting systemic inflammation, our study did not evaluate therapeutic interventions directly. The proposed value of TyG-guided anti-inflammatory strategies should therefore be considered exploratory, and further prospective studies are required to determine whether such approaches could improve NMIBC outcomes. Moreover, the high proportion of male patients (83.91%) may result in the underrepresentation of gender-specific biological factors like hormonal variations and lifestyle differences, potentially skewing the analysis of the TyG index’s relationship with NMIBC prognosis. Previous research has indicated that tumour biology and treatment responses in female NMIBC patients may differ from those in male patients (48). Given the low proportion of female patients in this study, the findings are likely to reflect the characteristics of the male cohort more prominently, which limits the generalizability of the results. Finally, further validation in larger, heterogeneous cohorts and prospective studies is necessary to confirm the clinical utility of the TyG index in routine practice.

## 5 Conclusion

This study demonstrates a strong association between the TyG index and the prognosis of NMIBC patients, exhibiting an inverted U-shaped relationship. It underscores the importance of maintaining the preoperative TyG index within an optimal range to effectively manage high-grade recurrence and disease progression in NMIBC. Additionally, the study highlights the role of TyG as a significant mediator of chronic systemic inflammation, suggesting its potential as a therapeutic target for intervention.

## Data Availability

The data analyzed in this study is subject to the following licenses/restrictions: Because the original data is still needed in other projects, it is temporarily unavailable, you can contact the corresponding author’s e-mail address to provide data. Requests to access these datasets should be directed to Junqi Wang, wangjq_68@163.com.

## References

[1] Siegel RL Giaquinto AN Jemal A. Cancer statistics, 2024. CA Cancer J Clin. (2024) 74:12–49. doi: 10.3322/caac.21820 38230766

[2] Fujii Y. Prediction models for progression of non-muscle-invasive bladder cancer: A review. Int J Urol. (2018) 25:212–8. doi: 10.1111/iju.13509 29247553

[3] Bree KK Shan Y Hensley PJ Lobo N Hu C Tyler DS. Management, surveillance patterns, and costs associated with low-grade papillary stage ta non-muscle-invasive bladder cancer among older adults, 2004-2013. JAMA Netw Open. (2022) 5:e223050. doi: 10.1001/jamanetworkopen.2022.3050 35302627 PMC8933744

[4] Sylvester RJ van der Meijden APM Oosterlinck W Witjes JA Bouffioux C Denis L. Predicting recurrence and progression in individual patients with stage Ta T1 bladder cancer using EORTC risk tables: a combined analysis of 2596 patients from seven EORTC trials. Eur Urol. (2006) 49:466–5; discussion 475-7. doi: 10.1016/j.eururo.2005.12.031 16442208

[5] Brausi M Witjes JA Lamm D Persad R Palou J Colombel M. A review of current guidelines and best practice recommendations for the management of nonmuscle invasive bladder cancer by the International Bladder Cancer Group. J Urol. (2011) 186:2158–67. doi: 10.1016/j.juro.2011.07.076 22014799

[6] Isharwal S Konety B. Non-muscle invasive bladder cancer risk stratification. Indian J Urol. (2015) 31:289–96. doi: 10.4103/0970-1591.166445 PMC4626912 26604439

[7] Silveira Rossi JL Barbalho SM Reverete de Araujo R Bechara MD Sloan KP Sloan LA. Metabolic syndrome and cardiovascular diseases: Going beyond traditional risk factors. Diabetes Metab Res Rev. (2022) 38:e3502. doi: 10.1002/dmrr.3502 34614543

[8] Qin Q Xu X Wang X Zheng X-Y. Obesity and risk of bladder cancer: a meta-analysis of cohort studies. Asian Pac J Cancer Prev. (2013) 14:3117–21. doi: 10.7314/apjcp.2013.14.5.3117 23803089

[9] Cantiello F Cicione A Salonia A Autorino R De Nunzio C Briganti A. Association between metabolic syndrome, obesity, diabetes mellitus and oncological outcomes of bladder cancer: a systematic review. Int J Urol. (2015) 22:22–32. doi: 10.1111/iju.12644 25345683

[10] Djiogue S Nwabo Kamdje AH Vecchio L Kipanyula MJ Farahna M Aldebasi Y. Insulin resistance and cancer: the role of insulin and IGFs. Endocr Relat Cancer. (2013) 20:R1–R17. doi: 10.1530/ERC-12-0324 23207292

[11] Inoue M Tsugane S. Insulin resistance and cancer: epidemiological evidence. Endocr Relat Cancer. (2012) 19:F1–8. doi: 10.1530/ERC-12-0142 22851686

[12] Szablewski L. Insulin resistance: the increased risk of cancers. Curr Oncol. (2024) 31:998–1027. doi: 10.3390/curroncol31020075 38392069 PMC10888119

[13] Montella M Di Maso M Crispo A Grimaldi M Bosetti C Turati F. Metabolic syndrome and the risk of urothelial carcinoma of the bladder: a case-control study. BMC Cancer. (2015) 15:720. doi: 10.1186/s12885-015-1769-9 26475132 PMC4609154

[14] Mirr M Skrypnik D Bogdański P Owecki M. Newly proposed insulin resistance indexes called TyG-NC and TyG-NHtR show efficacy in diagnosing the metabolic syndrome. J Endocrinol Invest. (2021) 44:2831–43. doi: 10.1007/s40618-021-01608-2 PMC8572197 34132976

[15] Guerrero-Romero F Simental-Mendía LE González-Ortiz M Martínez-Abundis E Ramos-Zavala MG Hernández-González SO. The product of triglycerides and glucose, a simple measure of insulin sensitivity. Comparison with the euglycemic-hyperinsulinemic clamp. J Clin Endocrinol Metab. (2010) 95:3347–51. doi: 10.1210/jc.2010-0288 20484475

[16] Adams-Huet B Zubirán R Remaley AT Jialal I. The triglyceride-glucose index is superior to homeostasis model assessment of insulin resistance in predicting metabolic syndrome in an adult population in the United States. J Clin Lipidol. (2024) 18:e518–24. doi: 10.1016/j.jacl.2024.04.130 38834412

[17] Kim YM Kim J-H Park JS Baik SJ Chun J Youn YH. Association between triglyceride-glucose index and gastric carcinogenesis: a health checkup cohort study. Gastric Cancer. (2022) 25:33–41. doi: 10.1007/s10120-021-01222-4 34355281

[18] Fritz J Jochems SHJ Bjørge T Wood AM Häggström C Ulmer H. Body mass index, triglyceride-glucose index, and prostate cancer death: a mediation analysis in eight European cohorts. Br J Cancer. (2024) 130:308–16. doi: 10.1038/s41416-023-02526-1 PMC10803806 38087039

[19] Qin G Sun Z Jin Y Ren X Zhang Z Wang S. The association between the triglyceride-glucose index and prognosis in postoperative renal cell carcinoma patients: a retrospective cohort study. Front Endocrinol (Lausanne). (2024) 15:1301703. doi: 10.3389/fendo.2024.1301703 38476671 PMC10927751

[20] Libby P. Inflammation during the life cycle of the atherosclerotic plaque. Cardiovasc Res. (2021) 117:2525–36. doi: 10.1093/cvr/cvab303 PMC8783385 34550337

[21] DeFronzo RA. Insulin resistance, lipotoxicity, type 2 diabetes and atherosclerosis: the missing links. The Claude Bernard Lecture 2009. Diabetologia. (2010) 53:1270–87. doi: 10.1007/s00125-010-1684-1 PMC2877338 20361178

[22] Adams-Huet B Jialal I. An increasing triglyceride-glucose index is associated with a pro-inflammatory and pro-oxidant phenotype. J Clin Med. (2024) 13:3941. doi: 10.3390/jcm13133941 38999506 PMC11242814

[23] Mollinedo F. Neutrophil degranulation, plasticity, and cancer metastasis. Trends Immunol. (2019) 40:228–42. doi: 10.1016/j.it.2019.01.006 30777721

[24] Sierko E Wojtukiewicz MZ. Inhibition of platelet function: does it offer a chance of better cancer progression control? Semin Thromb Hemost. (2007) 33:712–21. doi: 10.1055/s-2007-991540 18000800

[25] Zhou Y Li T Muheiyati G Duan Y Xiao S Gao Y. Triglyceride-glucose index is a predictor of the risk of prostate cancer: a retrospective study based on a transprostatic aspiration biopsy population. Front Endocrinol (Lausanne). (2024) 14:1280221. doi: 10.3389/fendo.2023.1280221 38260162 PMC10801031

[26] Liu T Zhang Q Wang Y Ma X Zhang Q Song M. Association between the TyG index and TG/HDL-C ratio as insulin resistance markers and the risk of colorectal cancer. BMC Cancer. (2022) 22:1007. doi: 10.1186/s12885-022-10100-w 36138391 PMC9503258

[27] Zhang J Yin B Xi Y Bai Y. Triglyceride-glucose index is a risk factor for breast cancer in China: a cross-sectional study. Lipids Health Dis. (2024) 23:29. doi: 10.1186/s12944-024-02008-0 38279158 PMC10811843

[28] Tarantino G Crocetto F Di Vito C Creta M Martino R Pandolfo SD. Association of NAFLD and insulin resistance with non metastatic bladder cancer patients: A cross-sectional retrospective study. J Clin Med. (2021) 10:346. doi: 10.3390/jcm10020346 33477579 PMC7831331

[29] Gill E Sandhu G Ward DG Perks CM Bryan RT. The sirenic links between diabetes, obesity, and bladder cancer. Int J Mol Sci. (2021) 22:11150. doi: 10.3390/ijms222011150 34681810 PMC8539374

[30] Wu X Wang S Lin L Jia X Hu C Qi H. Association between triglyceride glucose index and breast cancer in 142,184 Chinese adults: findings from the REACTION study. Front Endocrinol (Lausanne). (2024) 15:1321622. doi: 10.3389/fendo.2024.1321622 38904041 PMC11186986

[31] Vidal CM Alva-Ornelas JA Chen NZ Senapati P Tomsic J Robles VM. Insulin resistance in women correlates with chromatin histone lysine acetylation, inflammatory signaling, and accelerated aging. Cancers (Basel). (2024) 16:2735. doi: 10.3390/cancers16152735 39123463 PMC11311683

[32] Grandl G Wolfrum C. Hemostasis, endothelial stress, inflammation, and the metabolic syndrome. Semin Immunopathol. (2018) 40:215–24. doi: 10.1007/s00281-017-0666-5 PMC5809518 29209827

[33] Hotamisligil GS. The role of TNFalpha and TNF receptors in obesity and insulin resistance. J Intern Med. (1999) 245:621–5. doi: 10.1046/j.1365-2796.1999.00490.x 10395191

[34] Al-Daghri NM Wani K AlHarthi H Alghamdi A Alnaami AM Yakout SM. Sex-specific signature in the circulating NLRP3 levels of saudi adults with metabolic syndrome. J Clin Med. (2021) 10:3288. doi: 10.3390/jcm10153288 34362072 PMC8347773

[35] Shi H Kokoeva MV Inouye K Tzameli I Yin H Flier JS. TLR4 links innate immunity and fatty acid-induced insulin resistance. J Clin Invest. (2006) 116:3015–25. doi: 10.1172/JCI28898 PMC1616196 17053832

[36] Wellen KE Hotamisligil GS. Obesity-induced inflammatory changes in adipose tissue. J Clin Invest. (2003) 112:1785–8. doi: 10.1172/JCI20514 PMC297006 14679172

[37] Koenen TB Stienstra R van Tits LJ Joosten LAB van Velzen JF Hijmans A. The inflammasome and caspase-1 activation: a new mechanism underlying increased inflammatory activity in human visceral adipose tissue. Endocrinology. (2011) 152:3769–78. doi: 10.1210/en.2010-1480 21862623

[38] O’Brian D Prunty M Hill A Shoag J. The role of C-reactive protein in kidney, bladder, and prostate cancers. Front Immunol. (2021) 12:721989. doi: 10.3389/fimmu.2021.721989 34512646 PMC8429489

[39] Ravishankaran P Karunanithi R. Clinical significance of preoperative serum interleukin-6 and C-reactive protein level in breast cancer patients. World J Surg Oncol. (2011) 9:18. doi: 10.1186/1477-7819-9-18 21294915 PMC3045973

[40] Masson-Lecomte A Rava M Real FX Hartmann A Allory Y Malats N. Inflammatory biomarkers and bladder cancer prognosis: a systematic review. Eur Urol. (2014) 66:1078–91. doi: 10.1016/j.eururo.2014.07.033 25151017

[41] Briukhovetska D Dörr J Endres S Libby P Dinarello CA Kobold S. Interleukins in cancer: from biology to therapy. Nat Rev Cancer. (2021) 21:481–99. doi: 10.1038/s41568-021-00363-z PMC8173513 34083781

[42] Kaaks R Lukanova A. Energy balance and cancer: the role of insulin and insulin-like growth factor-I. Proc Nutr Soc. (2001) 60:91–106. doi: 10.1079/pns200070 11310428

[43] Lopez-Beltran A Blanca A Cimadamore A Montironi R Luque RJ Volavšek M. T1 bladder carcinoma with variant histology: pathological features and clinical significance. Virchows Arch. (2022) 480:989–98. doi: 10.1007/s00428-021-03264-6 PMC9033727 35122124

[44] Liu K Nicoletti R Zhao H Chen X Chiu PK-F Ng C-F. The potential benefits of concomitant statins treatment in patients with non-muscle-invasive bladder cancer. BJU Int. (2025) 135:88–94. doi: 10.1111/bju.16493 39257199

[45] Parada B Reis F Pinto Â Sereno J Xavier-Cunha M Neto P. Chemopreventive efficacy of Atorvastatin against nitrosamine-induced rat bladder cancer: antioxidant, anti-proliferative and anti-inflammatory properties. Int J Mol Sci. (2012) 13:8482–99. doi: 10.3390/ijms13078482 PMC3430246 22942715

[46] Nardelli C Aveta A Pandolfo SD Tripodi L Russo F Imbimbo C. Microbiome profiling in bladder cancer patients using the first-morning urine sample. Eur Urol Open Sci. (2023) 59:18–26. doi: 10.1016/j.euros.2023.11.003 38298766 PMC10829607

[47] Crupi E de Padua TC Marandino L Raggi D Dyrskjøt L Spiess PE. Circulating tumor DNA as a predictive and prognostic biomarker in the perioperative treatment of muscle-invasive bladder cancer: A systematic review. Eur Urol Oncol. (2024) 7:44–52. doi: 10.1016/j.euo.2023.05.012 37330413

[48] Dobruch J Daneshmand S Fisch M Lotan Y Noon AP Resnick MJ. Gender and bladder cancer: A collaborative review of etiology, biology, and outcomes. Eur Urol. (2016) 69:300–10. doi: 10.1016/j.eururo.2015.08.037 26346676

