# Supplementary material for: Inflammation as a key mediator: linking triglyceride-glucose index to prognosis in non-muscle-invasive bladder cancer
Source: Front Oncol. 2025 May 23;15:1545985. doi: 10.3389/fonc.2025.1545985 (PMC12141014; doi:10.3389/fonc.2025.1545985)
Supplement: Supplementary file 1 [file DataSheet1.zip › Supplementary Materials.docx]

**Supplementary Materials**

**Figures**

**
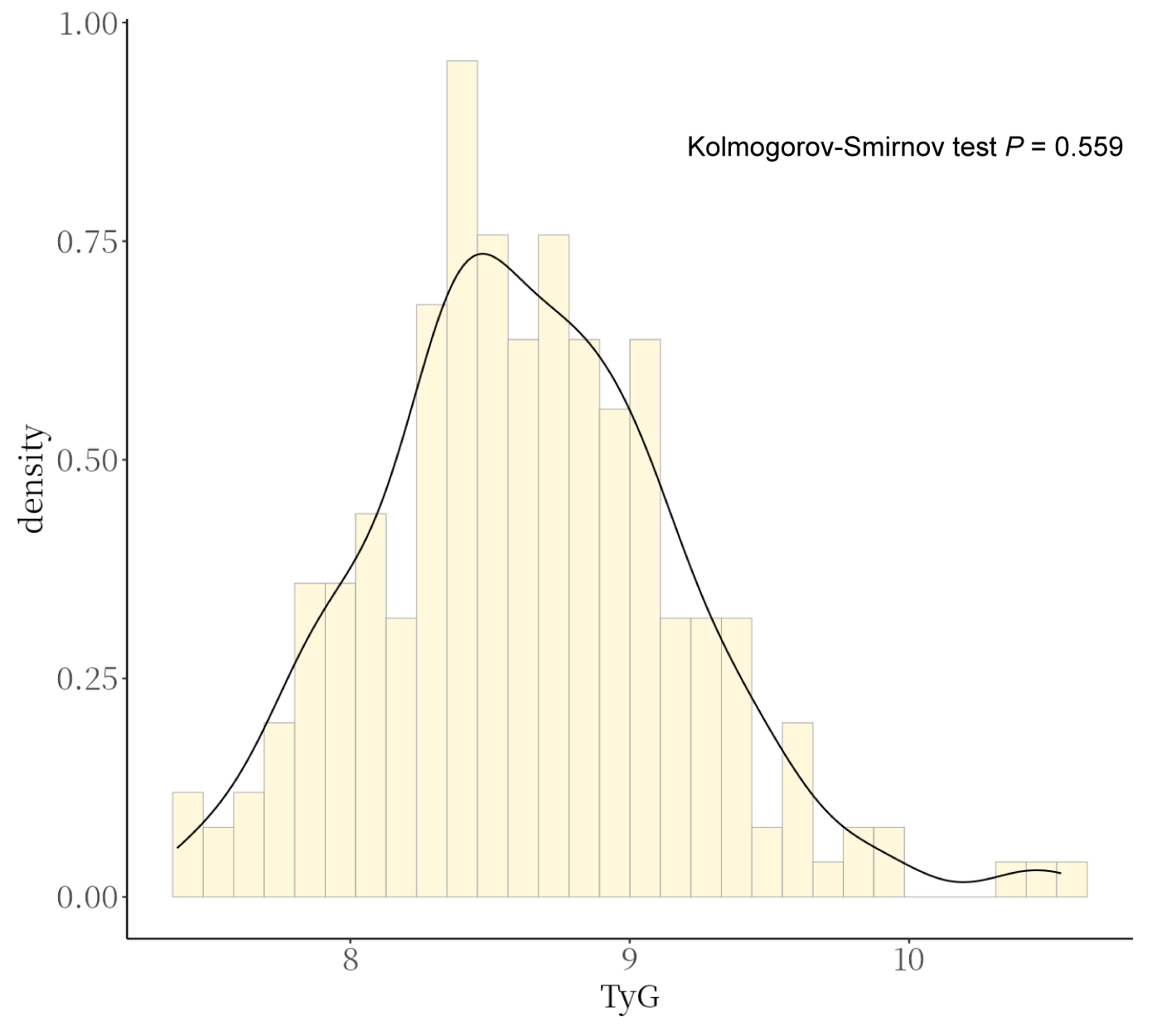
**

**Supplementary Figure 1. Distribution Pattern of the TyG Index**

**
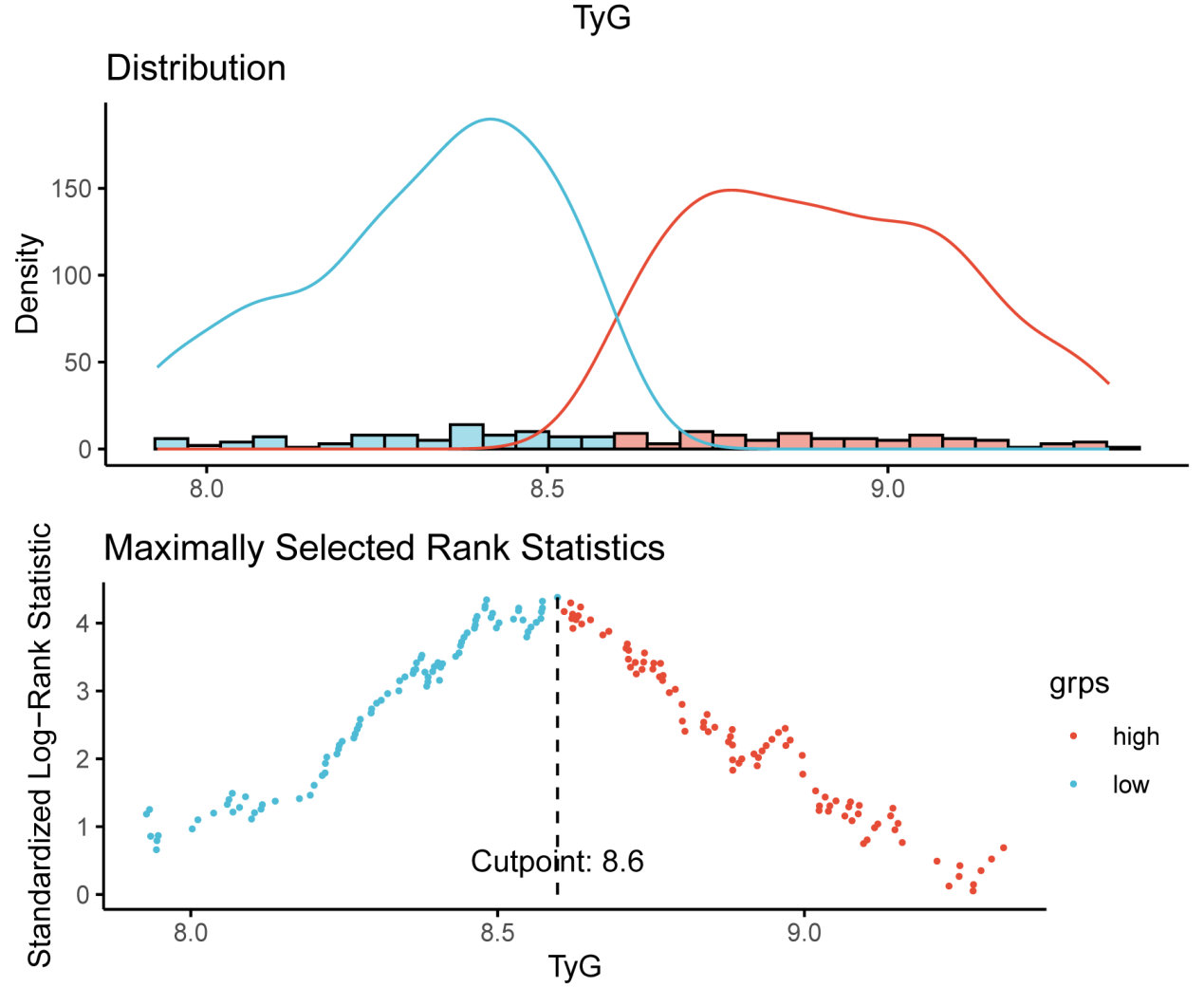
**

**Supplementary Figure 2. TyG Index Values Corresponding to the highest Youden Index**
